# Destination Impact! The Many Roads to Influencing Health System Change: A Response to Recent Commentaries

**DOI:** 10.34172/ijhpm.2020.77

**Published:** 2020-05-23

**Authors:** Mark Embrett, S Meaghan Sim, Katie Aubrecht, Ivy Cheng, Jonathan Lai, Rebecca Liu, Samiratou Ouedraogo, Margaret Saari

**Affiliations:** ^1^Health Program, Faculty of Arts and Science, St. Francis Xavier University, Antigonish, NS, Canada.; ^2^Nova Scotia Health Authority, Halifax, NS, Canada.; ^3^Healthy Populations Institute, Dalhousie University, Halifax, NS, Canada.; ^4^Department of Sociology, St. Francis Xavier University, Antigonish, NS, Canada.; ^5^Division of Emergency Medicine (DOM), University of Toronto, Toronto, ON, Canada.; ^6^Sunnybrook Health Sciences Center, Toronto, ON, Canada.; ^7^Canadian Autism Spectrum Disorders Alliance (CASDA), Toronto, ON, Canada.; ^8^Women’s College Hospital Institute for Health Systems and Virtual Care, Toronto, ON, Canada.; ^9^Dalla Lana School of Public Health, University of Toronto, Toronto, ON, Canada.; ^10^Department of Epidemiology, Biostatistics and Occupational Health, McGill University, Montréal, QC, Canada.; ^11^Institut National de Santé Publique du Québec (INSPQ), Montréal, QC, Canada.; ^12^SE Health, Markham, ON, Canada.

In our original article,^1^ we portrayed the perspectives of a group of trainees from the early months of the inaugural cohort of Canadian Institutes of Health Research’s (CIHR’s) Health System Impact (HSI) Fellowship, and in doing so we provided a framework for ‘Understanding the HSI Fellow as an Embedded Researcher’ and the various factors that may influence the HSI Fellowship experience. We are pleased that the article received perspectives from an international audience. Commentators compared the HSI Fellowship to similar programs in Australia^2^ and the United Kingdom^3^; others highlighted the historical challenges of these programs to make an impact^4,5^; another positioned integrated knowledge translation (iKT) at the centre of the fellowship experience.^6^ In response to these articles, we wish to: (1) address interpretations that were made regarding the role of the fellow; (2) emphasize two overlooked objectives of the fellowship (learning about and addressing health system challenges and professional development), thereby establishing that the fellowship and its impact is not ‘all about’ any one component; and (3) reflect on the commentators’ perspectives regarding tensions that exist between academic success and research impact. Our hope is that these responses will further illuminate the critical ways the HSI Fellowship is ‘driving change’ in the pursuit of a learning health system culture.

*Role of the fellow within the fellowship.* Several commentators built their arguments on, what we believe are, misinterpretations of the original article, which we will use this opportunity to address. Cassidy et al^6^ interpret our reasons behind using the term ‘driving change’ as positioning “the fellow as the central agent amidst the dual health system/academic environments with the goal of ‘driving change’ in the health system” (p. 455). Similarly, Rycroft-Malone and Langley^3^ choose fragments of two sentences, appearing several paragraphs apart, to argue “Sim et al describe the role of an HSI fellow as a ‘central agent’ who navigates the health system to become a ‘conduit for system-level change’” (p. 3). First, we wish to clarify the role of the fellow as the central agent. We position the fellow as a central figure in the fellowship in the description of the Framework for Understanding HSI Fellow as an Embedded Researcher. Our intention was to describe “the fellowship through a common lens to bring cohesion to the disparate experiences, both personally and within the organizations in which [fellows] were embedded” (p. 326). In the framework, the fellow is positioned alongside the academic and host organization, with the fellowship experience at the centre of the fellowship. Secondly, would like to address concerns regarding the misinterpretation that the fellow is driving change within the health system. It is evident from our article that “The *HSI Fellowship* is helping to ‘drive change’ and modernize the health system.” We positioned the fellows, their host organization, and their academic supervisor as partners in the pursuit of health system improvement and learning, and the fellowship as an impactful mechanism for system change. The lower part of the framework illustrates CIHR’s iKT approach of “a collaborative model of research, where researchers and knowledge users (those in the health system setting) work together to understand and address complex healthcare problems.”^7^

*Let’s not forget the training.* Our article was critiqued for limiting the role that iKT plays in supporting impact.^6^ This may be true as iKT was not the focus of our article. However, we also contend that the perspective of “it’s all about iKT”^6^ minimizes the “active and experiential learning”^8^ component of the fellowship that concern the fellows’ professional development and embedded training. The professional development component of the HSI fellowship supports the fellow in their development of both research and non-academic competencies^9^ in such areas as “negotiation and dialogue,” “change management” and “project management.”^9^ Moreover, we suggest that the competencies gained through HSI Fellowship-support (eg, leadership, negotiation, dialogue) are *critical antecedents for the effectiveness of iKT*. In this regard, the importance of fellows’ professional development as a component of the fellowship cannot be overlooked. This is also recognized among HSI Fellowship participants. An eDelphi study done with HSI fellows, host supervisors and academic supervisors unanimously agreed that criteria for a successful fellowship includes developing fellows’ core competencies, better knowledge of health system and policy, academic and research productivity as well as a complete HSI Fellowship project.^10^ Furthermore, both fellows and their supervisors saw improvement in all 10 HSI fellows’ core competencies by the 12-month mark.^11^ In our original article, we recognized the importance of this by stating that HSI Fellowship enables both personal and professional transformation and our conceptualization of the process of creating fellowship-specific outputs stated “most important is the intersection or co-production of outputs that are created as the host organization and the HSI fellow (with support from their academic supervisor) operate within this mutual learning space with best available evidence and practices at that moment” (p. 327).

*Comparative Learning.* Our commentary was fortunate to receive responses from several international researchers who compared the HSI Fellowship with their country’s embedded researcher training initiatives. Australia’s Embedded Health Management Academic^2^ and the UK’s National Institute for Health Research Knowledge Mobilisation Research Fellowship scheme^3^ are two pioneering approaches. While there are differences, a similar challenge among all programs is how to sustain and measure impact. Hunter^4^ takes a research specialization approach empathizing a more diverse and specialized research task force. Further, the trainee should not be expected to excel in every competency needed to optimize research utilization. Rather, researchers should be encouraged to specialize in one area of knowledge generation and translating: grant writing, teaching, or communicating with policymakers. McKee^5^ emphasizes the need for clear translation of research evidence in a timely, relevant manner. Embeddedness may increase the likelihood that this happens, but does not ensure it. Several innovative approaches that HSI Fellowship mentors are employing, not observed in other reports, include “providing the fellow with a committee of mentors within the organization, holding regular meetings with the fellow and both the health system and the academic supervisor, and leveraging their own network to expand the network and resources available to the fellow.”^12^ This practice can only help to further develop the fellows’ experiential learning, the relationships with both academic and knowledge user audiences, and to support greater opportunity for impact.

## Ethical issues

Not applicable.

## Competing interests

Authors declare that they have no competing interests.

## Authors’ contributions

All authors provided intellectual contribution to this article. SMS and JL drafted the article. All authors edited, provided written contributions, and reviewed the final version.

## Authors’ affiliations

^1^Health Program, Faculty of Arts and Science, St. Francis Xavier University, Antigonish, NS, Canada. ^2^Nova Scotia Health Authority, Halifax, NS, Canada. ^3^Healthy Populations Institute, Dalhousie University, Halifax, NS, Canada. ^4^Department of Sociology, St. Francis Xavier University, Antigonish, NS, Canada. ^5^Division of Emergency Medicine (DOM), University of Toronto, Toronto, ON, Canada. ^6^Sunnybrook Health Sciences Center, Toronto, ON, Canada. ^7^Canadian Autism Spectrum Disorders Alliance (CASDA), Toronto, ON, Canada. ^8^Women’s College Hospital Institute for Health Systems and Virtual Care, Toronto, ON, Canada. ^9^Dalla Lana School of Public Health, University of Toronto, Toronto, ON, Canada. ^10^Department of Epidemiology, Biostatistics and Occupational Health, McGill University, Montréal, QC, Canada. ^11^Institut National de Santé Publique du Québec (INSPQ), Montréal, QC, Canada. ^12^SE Health, Markham, ON, Canada.
